# MeXpose 2.0: A Comprehensive Toolkit for Single-Cell Spatial (Metall-)omics—Covering All Steps from Image Reconstruction to Integrative Analysis

**DOI:** 10.1021/cbmi.6c00070

**Published:** 2026-07-10

**Authors:** Gabriel Braun, Lyndsey Hendriks, Claude Molitor, Hoang Anh Nguyenová, Elisabeth Foels, Martin Schaier, Gunda Koellensperger

**Affiliations:** † Institute of Analytical Chemistry, Faculty of Chemistry, 27258 University of Vienna, 1090 Vienna, Austria; ‡ Vienna Doctoral School in Chemistry (DoSChem), 27258 University of Vienna, 1090 Vienna, Austria; § Institute of Inorganic Chemistry, Faculty of Chemistry, 27258 University of Vienna, 1090 Vienna, Austria

**Keywords:** image processing, bioinformatics, metallomics, open-source, single-cell

## Abstract

MeXpose 2.0 is a major update to our interactive workflow for comprehensive analysis of multiplexed imaging data, optimized for spatial metallomics. This release streamlined the existing pipeline by replacing third-party software with custom Jupyter notebooks for image reconstruction, preprocessing, and data extraction. Additional features such as limit-of-detection calculations, interactive region-of-interest annotations, cytometry-style gating, and integrated spatial statistics enhance both exploratory and quantitative analyses within a single, compact environment. MeXpose 2.0 is free and open-source, available at https://github.com/koellenspergerlab/mexpose under the MIT license. Additional test data are available at https://doi.org/10.5281/zenodo.20287869.

## Graphical abstract

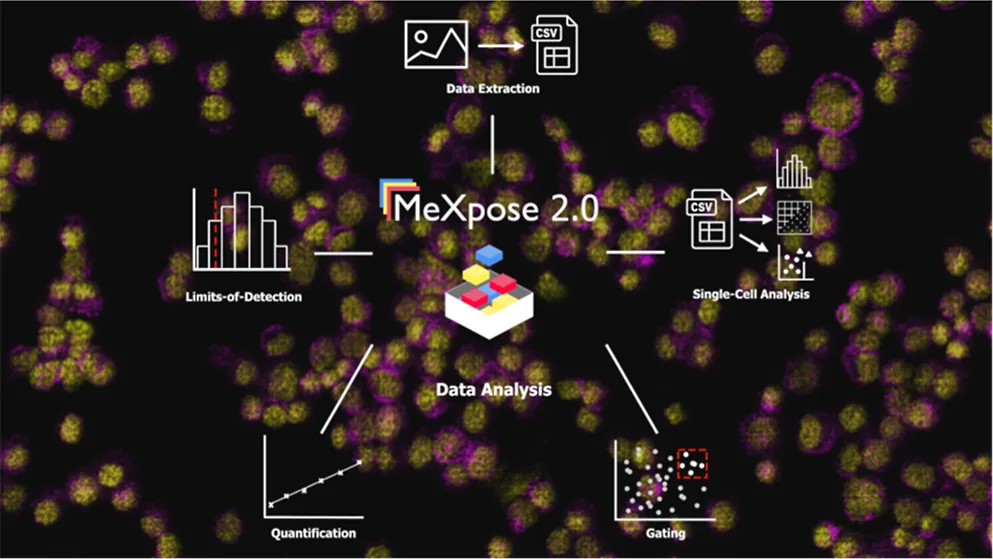

## Introduction

Spatial omics methodologies such as spatial transcriptomics and spatial proteomics have been lauded as the new emerging paradigm in life sciences.^1,2^ By preserving spatial context, these approaches enable researchers to investigate complex cellular phenotypes and interactions that are obscured in bulk analyses. Among the plethora of spatial omics modalities, many fall into the category of multiplexed imaging. These techniques often allow for routine imaging of more than 40 markers, which enables the identification of tissue and single-cell properties.^3,4^ Extracting and interpreting quantitative biological insights from these rich imaging data, however, remains challenging. The canonical analysis pipeline for multiplexed imaging data is comprised of multiple steps including preprocessing (image cleaning), segmentation, classification (phenotyping), and spatial analysis.^5^ Work on preprocessing and denoising segmentation algorithms and spatial analysis packages, among others, has facilitated and advanced the quality of multiplexed image analysis.^6–12^ Recently, novel approaches have attempted to circumvent some of the pitfalls of the standard analysis path by skipping segmentation and going straight to cell classification from the pixel-based data.^13,14^ Additionally, a variety of comprehensive workflows and packages have been developed with the intent to unify the individual steps. While we strongly support this incentive, these end-to-end solutions are often subject to a variety of shortcomings. Standalone software such as Cellprofiler and Fiji are well-known and used in the community.^15,16^ However, they require installation of plug-ins to achieve state-of-the-art end-to-end analysis, potentially making the workflow feel cumbersome. Software packages and command line tools such as Squidpy, Steinbock, MCMICRO, and SIMPLI are exceedingly powerful yet compromise on interactivity and/or require substantial technical knowledge to set up and run.^17–21^ Commercial solutions, though often polished and well-supported, come with financial, technical, and flexibility constraints—including licensing costs, version dependencies, and limited customizability due to proprietary design.

To address these limitations, we previously developed MeXpose, a software workflow designed to be a comprehensive, end-to-end multiplexed image analysis solution.^22^ MeXpose is specialized for, but not limited to, use on time-of-flight mass spectrometry (LA-ICP-TOFMS) data. This technique enables imaging of isotopes from *m*/*z* 7 to 272 including the ability to measure lanthanide markers as well as endogenous elements, leading to well over 200 image channels.^23^ MeXpose 1.0 combined in-house-developed tools with industry-standard software to provide a comprehensive workflow. Its functionalities encompass preprocessing, segmentation, clustering/phenotyping, and various visualization approaches. We focused on interactivity and modularity while retaining ease-of-use for researchers with basic knowledge of Jupyter Notebooks/Python, targeting the exploratory data analysis stage. To achieve this, we leveraged the interactivity provided by Jupyter Notebooks as well as selected functions of widely used graphical user interface (GUI)-based tools such as Fiji, Cellpose 2.0, and CellProfiler tied together in a Docker container.^15,16,24^ The pipeline offers both an interactive mode—allowing real-time control over image preprocessing, cell segmentation, and exploratory analysis—and a scalable, semiautomated mode for high-throughput studies, all within the same Docker-based environment. This combination of an intuitive interface with flexible automation and modularity helped lower the technical barrier for users while ensuring analysis could be tailored to different experimental scales. Furthermore, MeXpose incorporates quantitative calibration, enabling the direct measurement of absolute metal content per cell. To our knowledge, MeXpose is the only workflow for spatial metallomics data providing such a feature for images of tissues composed of multiple thousands of cells, making it uniquely useful in this realm.

Here, we present a major update to MeXpose. We have reinforced our efforts to provide an interactive, modular, and comprehensive end-to-end analysis pipeline. Therefore, we decided to focus this update on the interactive version of the workflow. This means we have enhanced the original workflow in several ways while keeping its functionalities centered around the core concepts of multiplexed image analysis. Key updates and features are illustrated in Figure 1 and include the following.

**Figure 1:**
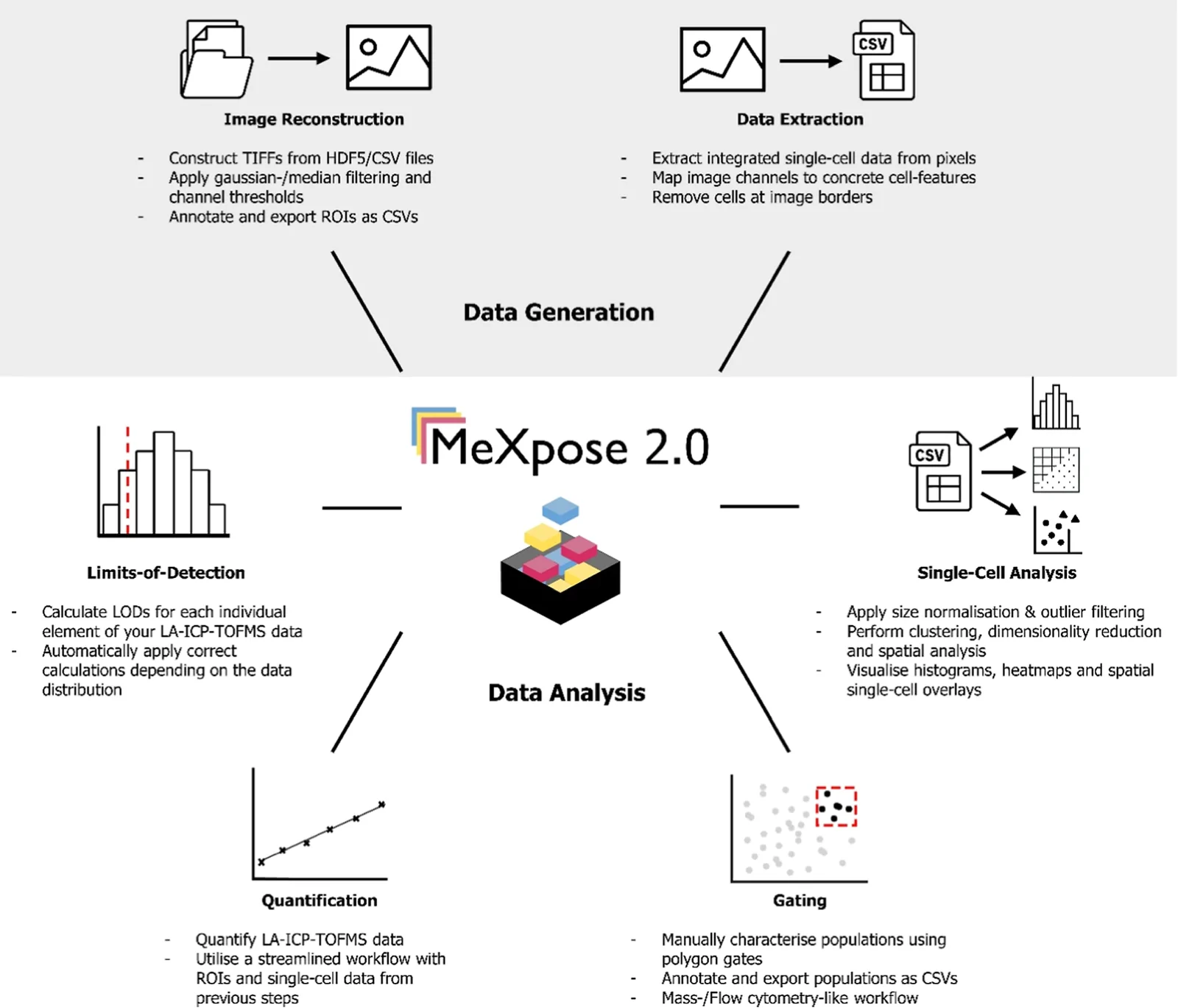
Overview of changes and new features introduced in MeXpose 2.0.

### Streamlined Workflow

- Replacement of third-party GUI tools with interactive Jupyter notebooks.
- Improved quantification workflow with interactive pixel-wise region-of-interest (ROI) annotation and export (Figure 2B).

### Added Functionality

Direct image reconstruction from raw laser and TOFMS data (Figure 2).
Interactive single-cell ROI annotation and export (Figure 3).
Spatial cell analysis using SCIMAP^12^ (Figure 4).
Limit-of-detection (LOD) calculation module (Figure 5).
Cytometry-style gating functionality.

We recommend the use of conda or mamba to create and manage a new virtual Python environment as we provide a compatible YAML file with all required dependencies.^25,26^

### New Features in MeXpose 2.0

We have reworked the internals of the MeXpose interactive workflow to be more streamlined and lightweight with a reduced reliance on third-party GUI software while retaining core functionality. Specifically, we have replaced Fiji (preprocessing) and CellProfiler (data extraction) with new Jupyter notebooks decorated with widgets as graphical user elements. This change removes the requirement to switch between multiple standalone GUI tools, thereby enhancing the workflow’s fluidity and intuitiveness.

Furthermore, MeXpose 2.0 eliminates the need for the use of commercial software, such as HDIP (Teledyne Photon Machines, Bozeman, MT, USA) or iolite (ESI Elemental Scientific Lasers, Bozeman, MT, USA), to reconstruct images from combined laser-shot coordinates and ICP-TOFMS integrated peak data. These data are produced by data acquisition software (TOFPilot, v2.15, TOFWERK, Thun, Switzerland; NuQuant, v1.2.8739.1, Nu Instruments Ltd., Wrexham, UK), which are bundled with the respective vendor devices. The TOFWERK data acquisition software, TOFPilot, outputs HDF5 files that contain the integrated peak data. A prerequisite for successful use of the MeXpose workflow is that the data are acquired using the laser ablation module in TOFPilot, which ensures that both laser coordinates and TOF data are aligned and stored together in a single file. Nu Instruments systems produce binary blob files (.inter), which must subsequently be processed in NuQuant to generate equivalent exported data sets with integrated signals and corresponding spatial coordinates. We have dedicated two vendor-specific notebooks (TOFWERK, Thun, Switzerland, and Nu Instruments, Wrexham, UK) to this function. These enable the user to select isotopes from their integrated peak data, embed these into a two-dimensional coordinate system, and finally plot the resulting image in an interactive way. Within the interactive visualization, we added (a) a histogram viewer of the recorded signal intensity per pixel; (b) basic preprocessing including thresholding and Gaussian and median filtering; (c) manual region-of-interest (ROI) annotation; (d) export of tabular pixel-based information on ROIs; and (e) TIFF image export of individual image channels (raw, preprocessed, or both) (Figure 2). Thresholding applies user-defined lower and upper intensity percentiles to suppress outliers. Gaussian and median filtering are provided as preprocessing options to improve image quality and reduce noise. Gaussian filtering smooths the image by convolution with a Gaussian kernel, thereby reducing local intensity variation. Median filtering replaces each pixel with the median value within a local neighborhood, which can be effective for removing impulse noise while preserving object boundaries.^27,28^ By including these functionalities, we facilitate vendor-agnostic data support for downstream data analysis. While the first step of the workflow typically is to reconstruct TIFF images from raw ICP–TOFMS data using the aforementioned manufacturer-specific notebooks, the pipeline can also be run with pre-existing TIFF files from other imaging techniques, such as fluorescence microscopy. Supported formats include, among others, TIFF, OME.TIFF, and BIGTIFF and are handled by the imageio Python library.^29^

**Figure 2:**
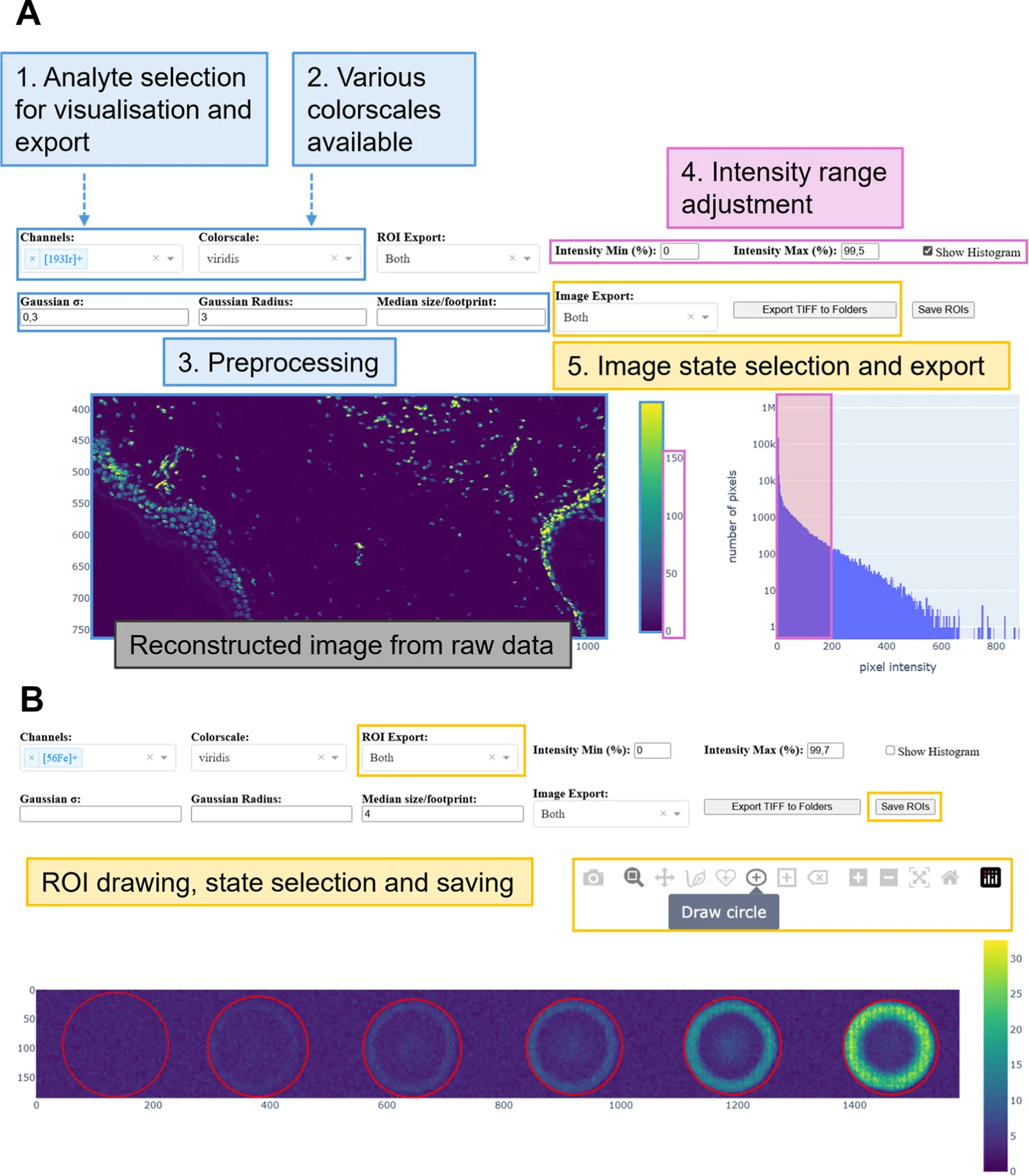
Screenshot of the image reconstruction module. (A) A reconstructed image from raw HDF5 data showing the cell nuclei of a skin cross-section stained with a DNA-intercalator (channel [193Ir]+). The intensity range was reduced to 99.5% of the maximum intensity, which is reflected in the red overlaid area in the histogram. A Gaussian filter with σ = 0.3 and radius = 3 was applied. (B) A reconstructed image from raw HDF5 data showing the iron (channel [56Fe]+) distributions within 6 gelatin droplet standards. Regions of interest (red circles) are interactively drawn on the image. The intensity range was reduced to 99.7% of the maximum intensity, and the histogram was toggled off. A median filter with (footprint -) size = 4 was applied. Colorbars in both panels indicate image channel intensity expression in counts per pixel. Image/ROI state selection dropdown menus allow exporting of “Raw”, “Processed”, or “Both” states of the data.

Following image reconstruction and data extraction, segmented cells are exported as a CSV, each row representing a cell with a cell label (*cell_label*), centroid coordinates (*Center*_*Y*, *Center*_*X*), area (*Area*), and diameter (*Diameter*) in pixels, and summed isotope intensities contained in the columns. The centroid specifies the center of a cell’s position in absolute image coordinates, while the diameter provides a pixel-based estimate derived from the segmented cell area. Building on this output, the interactive single-cell analysis stage was extended to support manual ROI annotation and export (Figure 3). This feature employs an image channel as the background, overlaying a segmentation map that is color-coded according to marker intensity or cluster affiliation. It is then possible for users to select and export segmented objects as tabular data by drawing annotations directly on the segmentation mask. Regions of interest can then be utilized in downstream analyses, including spatial distance and co-occurrence analysis between cell types.

**Figure 3:**
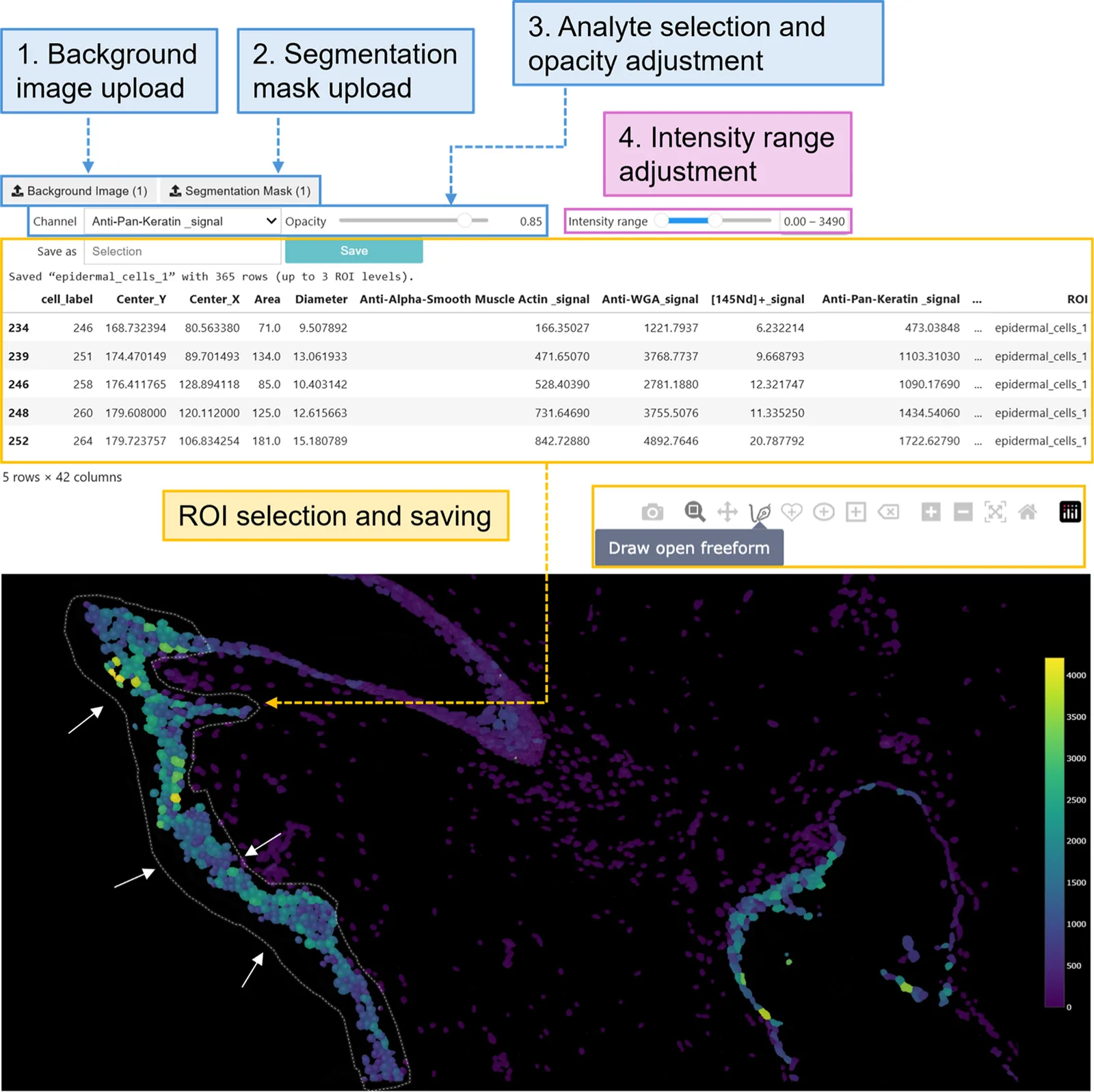
Screenshot of a cell mask intensity overlay and dataframe head of a saved region of interest (ROI) (white dotted line with white arrows). Cells on the outer perimeter of the skin cross-section were selected based on their pan-cytokeratin expression and labeled as “epidermal_cells_1”. Tabular data shows single-cell characteristics of the selected ROI (morphological features, marker expression). The colorbar displays pan-cytokeratin expression in signal intensity (counts) per cell.

The addition of these features specifically was achieved through the integration of the SCIMAP library, developed by Nirmal and Sorger, 2024.^12^ By wrapping the primary options of SCIMAP’s *spatial_distance* and *spatial_interaction* functions with Jupyter widgets, the workflow remains intuitive and interactive while leveraging SCIMAP’s computations and excellent visualizations (Figure 4). This allows the user to gain information about the likelihood of certain phenotypes interacting with each other (*spatial_interaction*) as well as the distance between individual cell types (*spatial_distance*).

**Figure 4:**
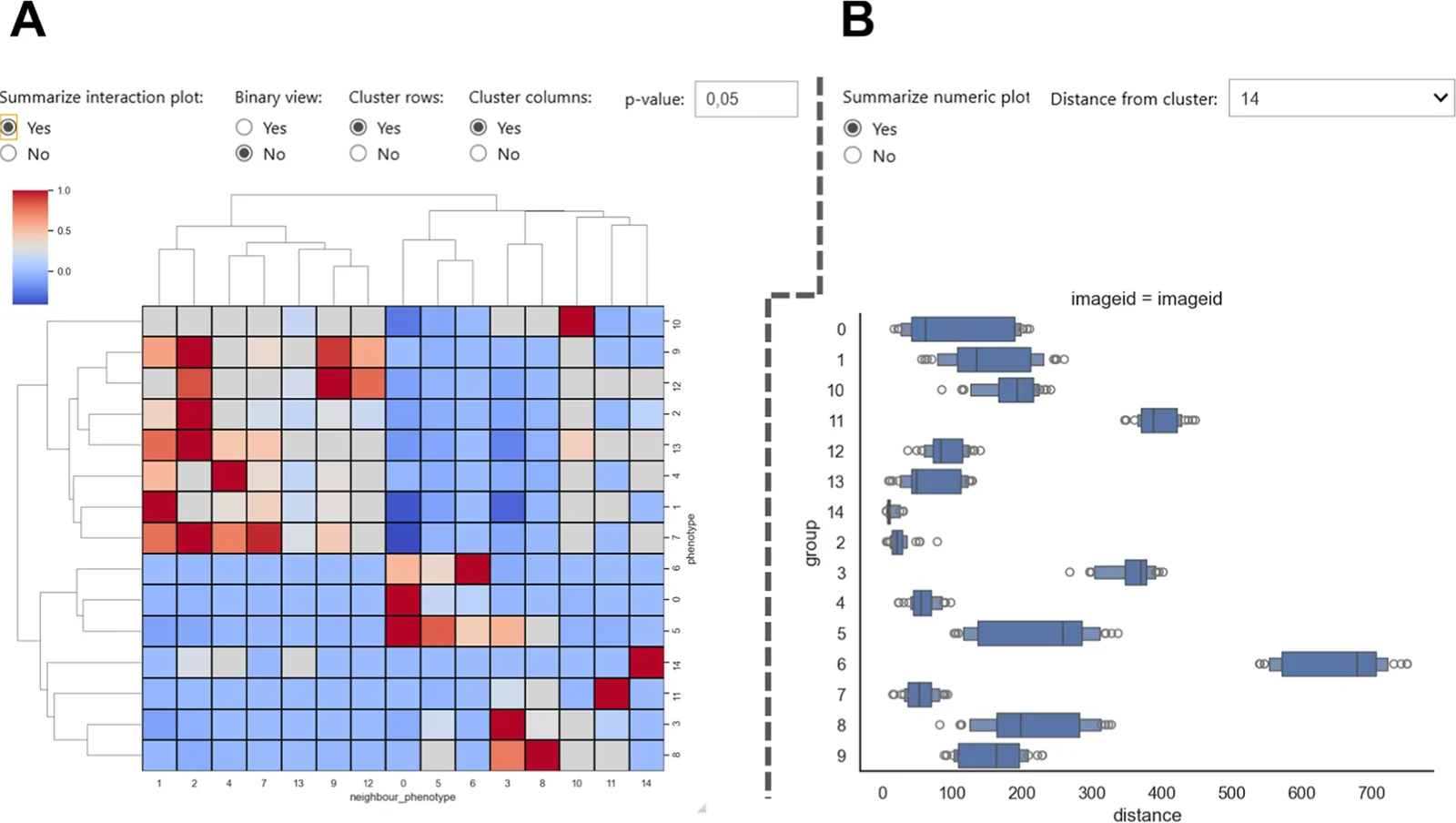
SCIMAP-based spatial analysis of cell type interactions and distances. (A) A heatmap showing the likelihood of spatial interaction between previously identified clusters (cell types) in the data. Dendrograms are enabled by setting “Cluster rows” and “Cluster columns” to “Yes”. The significance level to determine interaction is set to *p* = 0.05. (B) A numerical distance plot showing distances of identified clusters from cluster_14. Distances are shown in pixels on the *X*-axis; identified clusters are shown on the *Y*-axis.

A distinctive feature of the initial MeXpose release was the ability to perform absolute quantification of cellular metal contents using gelatin microdroplet standards.^30^ Quantification poses an integral aspect of reliable and reproducible LA–ICP–TOFMS data generation. To extend this incentive, MeXpose 2.0 provides a limit of detection (LOD) calculation module specifically for LA–ICP–TOFMS data.

Using a bootstrapping approach to sample a gelatin blank, the distribution of an isotope is analyzed (Figure 5). Rather than calculating the LOD for individual pixels, this method enables the calculation of LODs for regions of a specified size/pixel count such as the average size of a cell. Users can set a region size, as well as the number of bootstrap iterations. For each iteration, a set ,number of pixels is randomly sampled from a blank standard, and the corresponding pixel intensities (counts) are summed to generate a bootstrap distribution of region-level blank signals. This accounts for the heterogeneity of gelatin standards and reflects region- or cell-sized summed signals rather than averaged per-pixel intensities. The mean and standard deviation of these bootstrap sums are then used for calibration and LOD estimation. Depending on the observed distribution and a user-defined threshold value, which is implemented to prevent false assumptions of normal distributions at low intensity counts, the correct LOD formula is selected. For Gaussian distributions, a 3σ approach is applied, while for compound Poisson distributions, a Poisson approximation is used.^31,32^ For more details on the theoretical background behind this approach, refer to Foels et al.^33^ Upon calculation, results and logs are saved in tabular and text format, respectively, and can also be inspected visually by showing histograms and quantile–quantile plots.

**Figure 5:**
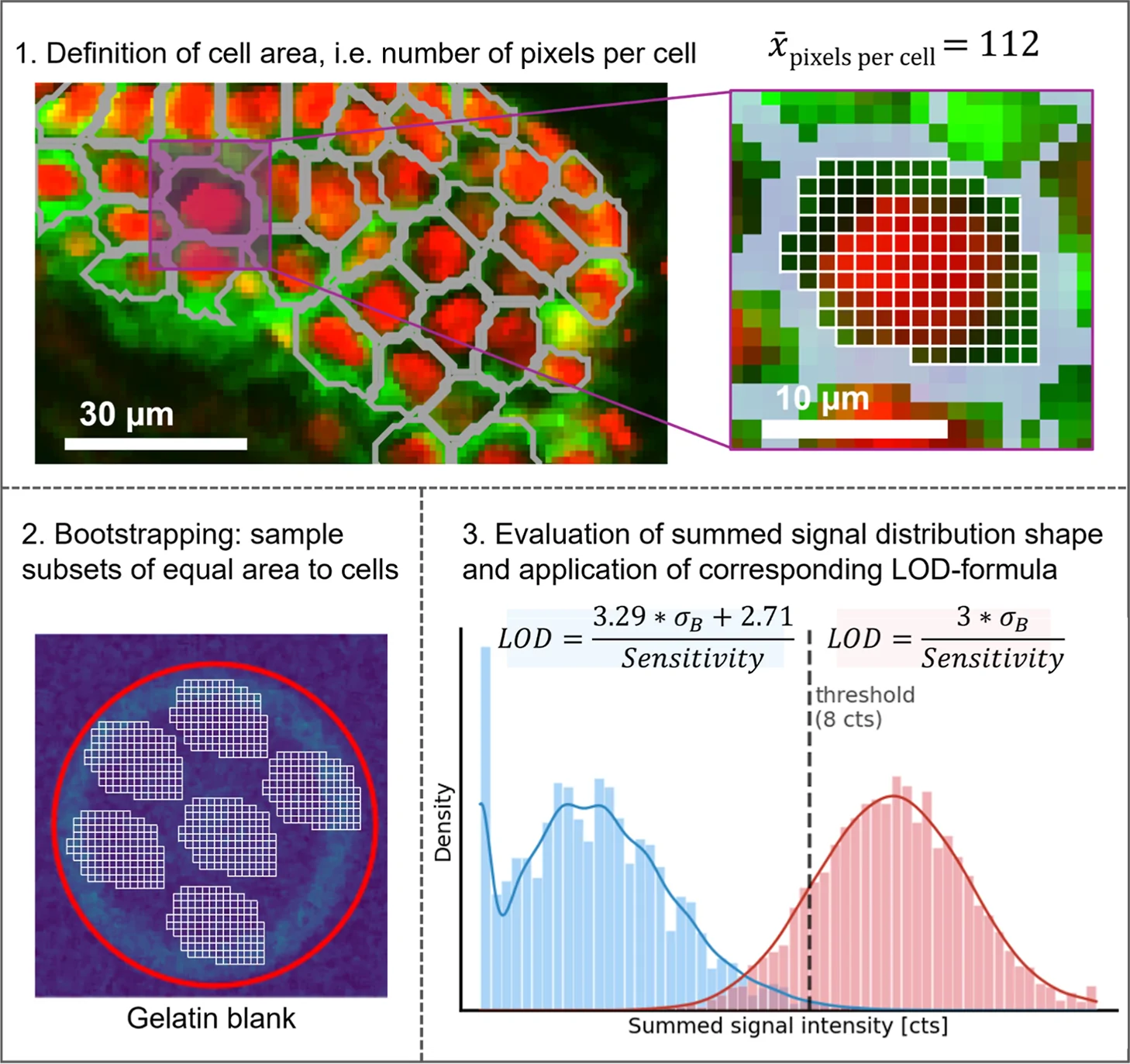
Schematic of the limit-of-detection (LOD) calculation procedure based on the same skin data set. Step 1 shows the determination of average cell size in a cross section of skin tissue on a stack of two channels, [193Ir]+ (red) and [153Eu]+ (green), with an overlaid segmentation mask generated in Cellpose-SAM (gray). Step 2 visualizes the bootstrapping process, drawing pixel populations from a blank gelatin droplet standard. Step 3 uses these bootstrap resamples to generate a distribution of summed region-level blank signals. Depending on the resulting distribution of these sums, either a 3σ approach (red) or a Poisson approximation (blue) is used to calculate the corresponding LOD. The smooth curves overlaid over the two distributions are kernel density estimations (KDEs), computed from the sampled bootstrap sums to provide an estimate of the underlying probability density.

Finally, we developed a gating module, enabling users to implement a manual hierarchical gating strategy for the effective identification of cell populations, analogous to that of canonical flow/mass cytometry data analysis. Single-cell data are shown as a two-dimensional scatter plot, plotting two channels against one another. Users can draw, label, and save polygon gates to isolate specific populations. Additionally, a channel-specific thresholding/clipping function has been implemented for single-cell outlier filtering. Channels can be selected and clipped to user-defined upper and lower boundaries by setting all exceeding values to their respective limits (Figure 6). Alongside the aforementioned major changes, minor contributions and revisions including (i) code refactoring, (ii) user interface improvements, (iii) extended support for image formats, and (iv) extended testing and support for various laser scanning modes have been incorporated into MeXpose 2.0.

**Figure 6:**
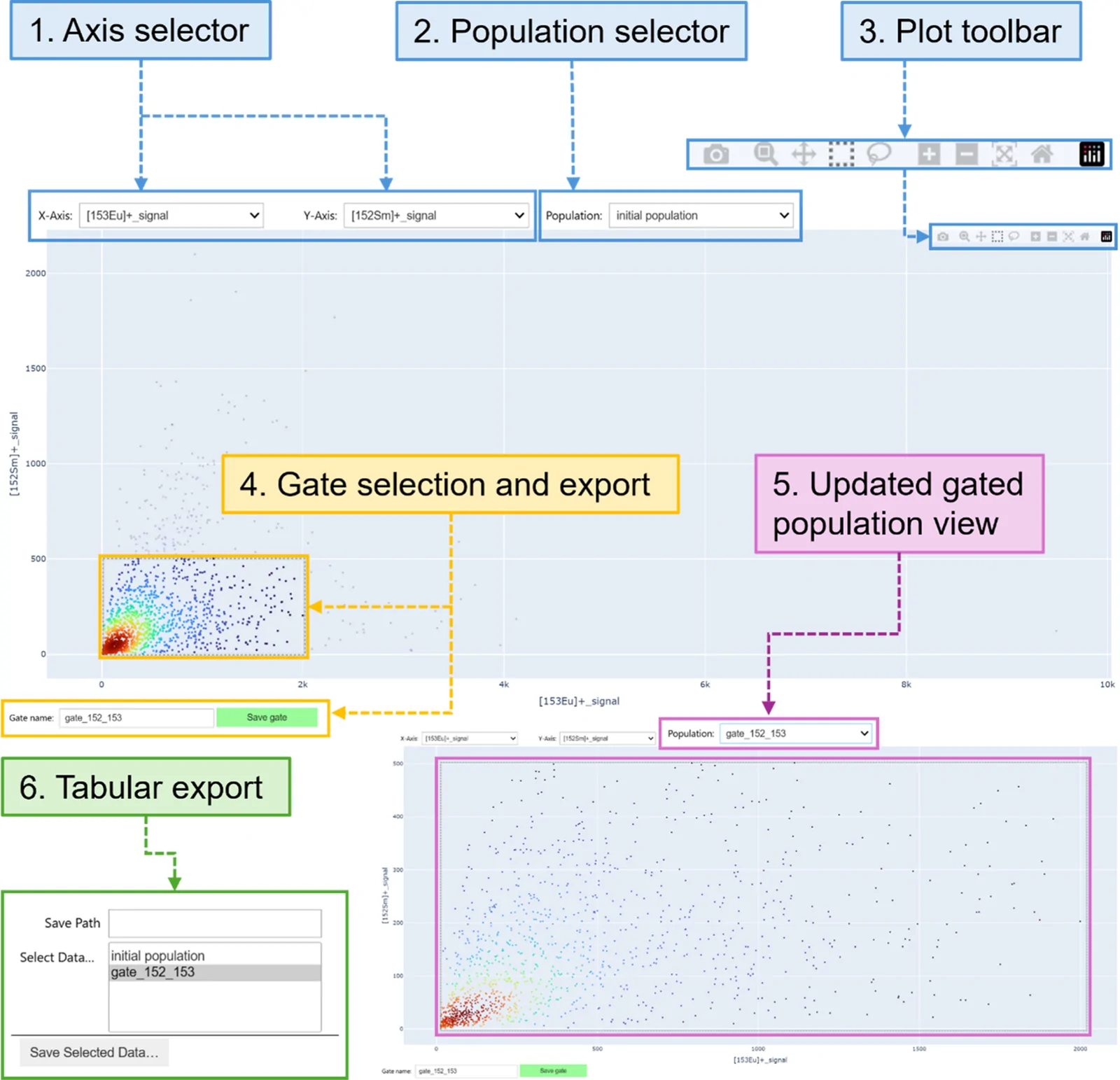
Screenshots of the gating Jupyter notebook. (1) Axis selectors set to [153Eu]^+^ (*x*-axis, cell membrane marker) and [152Sm]^+^ (*y*-axis, CD45 marker). (2) Population selector, initially showing the initial population and updated as gates are saved. (3) Interactive plot toolbar with lasso/box selection, zoom, and figure export. (4) Gate save controls. (5) Updated interface after gate saving. (6) Tabular export of saved populations.

## Conclusion

The changes made to MeXpose 2.0 further facilitate the exploratory and quantitative data analysis of LA-ICP-TOFMS and potentially other multiplexed imaging data (tested on fluorescence imaging data). We provide a lightweight, yet powerful workflow based on interactivity, visualization, and modularity, consolidating most steps into JupyterLab notebooks to replace multiple separate GUIs and minimize the learning curve. By offering a free and open-source alternative to proprietary image analysis software, we hope to contribute to the creation of high-quality insights and analyses from multiplexed imaging data. Looking forward, we strive to continuously improve and extend MeXpose, including integration with the Anndata framework, to ensure future relevance and facilitate compatibility with other projects.^30^

## Methods

Chemical reagents and immunostaining of used skin tissue sections, as well as LA–ICP–TOFMS analysis, data acquisition, and processing, are described in the original MeXpose publication.^22^ Anaconda (Anaconda Inc., v23.4.7) was used to manage Python versions and libraries. A YAML file is provided with required dependencies, including versions, at https://github.com/KoellenspergerLab/MeXpose/blob/main/mexpose_v2.yaml.

## Data Availability

The Supporting Information is available on Zenodo as a ZIP archive at https://doi.org/10.5281/zenodo.20287869. It includes PDF files with installation and usage instructions, a workflow overview graphic, the conda/mamba environment file (YAML), and a JupyterLab configuration file for large-file upload handling (PY). Further, the archive contains a test data set containing a text file for data description and the HDF5 input data file, as well as a generated single-cell table, standards summary table, and analyte amount table for each gelatin droplet, antibody panel, and prereconstructed TIFF image files.
